# Noncoding RNAs in Vascular Aging

**DOI:** 10.1155/2020/7914957

**Published:** 2020-01-04

**Authors:** Qidong Cao, Jiuping Wu, Xiaoli Wang, Chunli Song

**Affiliations:** ^1^Department of Cardiology, The Second Hospital Affiliated to Jilin University, Chang Chun, Jilin, China; ^2^Department of Spinal Surgery, The Second Hospital Affiliated to Jilin University, Chang Chun, Jilin, China; ^3^Department of Pneumology, Qingdao Women and Children's Hospital, Qingdao, Shandong, China

## Abstract

Increases in age are accompanied by vascular aging, which can lead to a variety of chronic diseases, including atherosclerosis and hypertension. Noncoding RNAs (ncRNAs) have become a research hotspot in different fields of life sciences in recent years. For example, these molecules have been found to have regulatory roles in many physiological and pathological processes. Many studies have shown that microRNAs (miRNAs) and long ncRNAs (lncRNAs) also play a regulatory role in vascular aging. Endothelial cells (ECs) and vascular smooth muscle cells (VSMCs) are important components of blood vessels, and the senescence of both cell types promotes the occurrence of vascular aging. This review provides a contemporary update on the molecular mechanisms underlying the senescence of ECs and VSMCs and the regulatory role of miRNAs and lncRNAs in this process.

## 1. Introduction

Cardiovascular disease is a common cause of death among the elderly. The mortality rate due to heart disease and stroke is known to increase exponentially with increased age, accounting for more than 40% of the total deaths of 65 to 74-year-old patients and nearly 60% of the total deaths of patients over 85 years old. Vascular aging is an independent risk factor for age-related diseases, especially cardiovascular diseases such as atherosclerosis, hypertension, and stroke, which are characterized by increased vascular stiffness and pulse wave velocity (PWV), enlarged lumens, and decreased vascular elasticity based on functional and morphological assessments [1, 2]. It is clear that vascular aging increases the risk of developing cardiovascular diseases.

It is accepted that 1–2% of the human genome is protein-coding, while the remaining 98% is mostly transcribed into RNA with no or minimal protein-coding potential, known as noncoding RNA (ncRNA). Studies suggest that these molecules act as key regulators in many biological processes including gene expression, cell cycle control, apoptosis, cell differentiation, chromatin remodeling, and epigenetic modifications [3, 4]. ncRNAs include “housekeeping” RNAs such as ribosomal RNA (rRNA) and transfer RNA (tRNA), as well as regulatory RNAs. Regulatory RNAs are categorized into small ncRNAs, which are shorter than 200 nt, and long ncRNAs (lncRNAs; > 200 nt) according to their transcript length. Small regulatory ncRNAs include microRNAs (miRNAs), PIWI-interacting RNAs (piRNAs), and endogenous small interfering RNAs (endo-siRNAs). Among them, a large number of studies have focused on the regulatory role of miRNAs and lncRNAs in the process of vascular aging.

Vascular smooth muscle cells (VSMCs) and endothelial cells (ECs) are the main components of blood vessels, and senescence in these cell types is thought to contribute to vascular aging and age-related diseases. Cell senescence is affected by many pathological and clinical factors, such as inflammation and diabetes. Inflammation plays an important role in cell senescence; some cytokines (such as nuclear factor-*κ*B (NF-*κ*B), CCAAT-enhancer-binding protein *β* (C/EBP*β*), and signal transducer and activator of transcription 3 (STAT3)) promote cell senescence [5]. In addition, it was found that hyperglycemia promotes endothelial cell senescence, and some miRNAs have been proven to play a role in diabetes mellitus and its vascular complications [6, 7]. In addition, hyperglycemia also promotes myocardial aging by activating miR-34a [8]. The mechanism by which these pathophysiological factors lead to vascular aging is an interesting topic of research.

Many studies have found that miRNAs and lncRNAs regulate the occurrence and development of vascular aging, leading to vascular aging-related dysfunction and diseases. Thus, it is necessary to develop clinical strategies for delaying vascular aging by targeting ncRNAs. Here, we review current research on the role of miRNAs and lncRNAs in EC and VSMC senescence. Furthermore, the major miRNAs and lncRNAs that influence senescence of ECs and VSMCs are introduced, and their roles in controlling vascular aging are discussed.

## 2. Senescence of ECs and VSMCs

Cell senescence can be defined as cell cycle arrest accompanied by the depletion of replication potential [9]. Senescent cells are characterized based on morphology (vacuolation, flattening cells) and the expression of specific markers such as senescence-related *β*-galactosidase (SA*β*G) [10]. Cell senescence has been categorized into two processes based on its causes: replicative senescence (RS) and stress-induced premature senescence (SIPS). Normal somatic cells cannot maintain their replicative capacity indefinitely, and thus, they eventually enter a state of growth arrest [11]. When cells are cultured *in vitro*, the telomeres shorten gradually and eventually reach the Hayflick limit, triggering the DNA damage response (DDR) and RS [12]. In turn, exposure to angiotensin II, oxidative stress, and mitochondrial and DNA damage can induce cellular senescence, which is referred to as SIPS [13].

Telomeres are repetitive TTAGGG DNA sequences located at the end of chromosomes that protect DNA from damage. Telomerase, a telomeric repeat synthase, is used to maintain the telomere length. Mechanically, critical telomere shortening is thought to trigger the onset of cellular senescence, and telomerase activity regulates cell proliferation in normal somatic cells by lengthening the telomere, or via telomere length-independent mechanisms [14]. Human ECs and VSMCs exhibit telomerase activity activated by mitogenic stimuli via a protein kinase C-dependent pathway [15]. However, the activity of telomerase declines with aging because of a decrease in the expression of telomerase reverse transcriptase (TERT), leading to telomere shortening and cellular senescence [16, 17]. One study found that the addition of NO to ECs rescued the replicative reduction of telomerase activity [18].

RS and SIPS are mediated by two distinct and partially intersecting pathways [19]. RS involves the p53 factor and its immediate transcriptional target p21, whereas SIPS involves p16 and retinoblastoma protein (pRB). Both p16 and p21 induce cell cycle arrest through the inhibition of cyclin-dependent kinases (CDKs), resulting in pRB activation [19]. The function of EC gradually declines with age, resulting in the progression to a systemic inflammatory state known as “inflammatory aging” [20]. In this state, cells exhibit a characteristic senescence-related secretory phenotype, characterized by high levels of matrix metalloproteinases (MMPs) and inflammatory cytokines [21]. Endothelial dysfunction induced by EC senescence includes the impairment of endothelium-dependent vasodilation, angiogenesis, and the antithrombotic properties of the endothelium [22]. Both NO production and endothelial NO synthase activity are reduced in senescent human vascular ECs, which show increased vasoconstrictive activity [23, 24]. In addition, NO inhibits platelet aggregation and blocks neutrophil/monocyte adhesion to ECs [25, 26]. However, NO depletion can lead to the dysfunction of vascular homeostasis and the development of hypertension, thrombosis, and atherosclerosis [27, 28].

VSMCs also play a key role in vascular aging and contribute to the occurrence and development of atherosclerosis [29]. In addition, VSMC senescence increases the vulnerability of atherosclerotic plaques [30]. VSMCs change from a contractile state to a synthetic one during vascular impairment, hypertension, or atherosclerosis [31]. miRNAs have been identified as key regulators of VSMC biology. For example, it was found that reduced miR-23b activity induces phenotypic switching in VSMCs after vascular injury [32]. Similarly, phenotypic switching of VSMCs is also common during vascular aging, making the cardiovascular system prone to disease even in the absence of traditional risk factors [33]. Senescence phenotypes also include a loss of VSMCs in media and markers of premature senescence including increased Sa*β*G expression, reduced telomere lengths, and a decrease in the number of Ki67-positive cells [34]. The loss of VSMCs can also lead to the formation of areas of mucoid degeneration, which play a role in chronic aneurysm and acute dissection associated with aging [35]. The overexpression of osteoblast-related genes and proteins (including RUNX-2, alkaline phosphatase (ALP), collagen I, and BMP-2) in senescent VSMCs leads to partial osteoblast transdifferentiation and vascular calcification [36]. Moreover, the increase in stiffness in the aging aorta is related to changes in the mechanical properties of VSMCs. Further, *β*1-integrin and *α*-smooth muscle actin are thought to be the main factors contributing to increased VSMC stiffness during aging [37]. Therefore, both EC and VSMC senescence can lead to vascular aging and related diseases including hypertension, atherosclerosis, aneurysm, and vascular dissection.

## 3. miRNAs and lncRNAs

miRNAs are short (∼22 nt) single-stranded ribonucleic acid molecules that bind the 3′-untranslated region (UTR) of a target gene, preventing the translation or promoting the degradation of the gene at the posttranscriptional level, thereby negatively regulating expression [38]. miRNAs begin with the transcription of primary RNA and are processed by RNase III DROSHA into 70 nt stem-loop transcripts, known as precursor- (pre-) miRNAs. Pre-miRNAs are then exported to the cytoplasm and processed by RNase III DICER1 into a mature ~22 nt double-stranded miRNA [39, 40]. One strand binds Argonaute (AGO) to form an RNA-induced silencing (RISC) complex when the double strands are unraveled [38]. miRNAs play a role in many biological processes including development, cell proliferation and differentiation, apoptosis, and immune regulation by negatively regulating the expression of target genes in cells [41]. In addition, miRNAs are also involved in the occurrence and development of many diseases, such as coronary heart disease, myocardial infarction, and heart failure, and can therefore be used as disease markers [42].

lncRNAs are broadly defined as ncRNA molecules longer than 200 nt. Most are transcribed by RNA polymerase II, and thus, they have a 5′-methylguanosine cap and a 3′-poly(A) tail structure, but lack the ability to encode proteins [43]. lncRNAs exist in many subcellular structures; however, initial studies have shown that they tend to be located in the nucleus and can activate or inhibit gene expression by introducing chromatin-modifying enzymes at specific genomic sites [44]. lncRNAs can also be used as a bait to separate transcription factors from genomic targets and inhibit gene transcription [45]. A substantial population of lncRNAs also exists in the cytoplasm to regulate mRNA and protein stability and translation, as well as function as miRNA sponges [45]. lncRNAs participate in many biological processes, such as cell proliferation, morphogenesis, pluripotency, development, neuronal processes, and gametogenesis, through transcriptional and posttranscriptional activity [46].

## 4. miRNAs and lncRNAs in Vascular Aging

**Figure 1:**
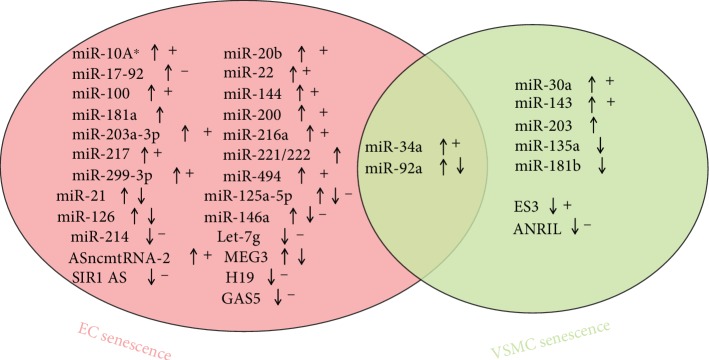
miRNAs and lncRNAs in the senescence of endothelial cells (ECs) and vascular smooth muscle cells (VSMCs). (↑) and (↓) indicate increased and decreased expressions, respectively, during senescence. (+) and (-) indicate the promotion or inhibition of senescence, respectively, by miRNA or lncRNA.

miRNAs and lncRNAs participate in many physiological and pathological processes, including vascular aging. The role of miRNAs and lncRNAs on senescence in ECs and VSMCs affects the process of vascular aging (Figure 1).

### 4.1. miRNAs and EC Senescence

ECs play an important role in vascular physiology; EC senescence leads to dysfunction and promotes the occurrence of diseases related to vascular aging. A senescent EC phenotype is associated with increased apoptosis, reduced endothelial nitric oxide synthase (eNOS) production, and inflammation. Many miRNAs are involved in the regulation of EC senescent phenotypes (Table 1). For example, senescent human aortic ECs (HAECs) exhibit reduced expression of proliferation-stimulating or apoptosis-suppressing miRNAs such as miR-21, miR-214, and miR-92a; increased eNOS-suppressing molecules including miR-221 and miR-222; reduced miR-126, which represses inflammation; and increased miR-125b, which stimulates inflammation. Further, the development of a senescent arterial EC phenotype is characterized by reduced cell proliferation, enhanced apoptosis, and inflammation; additionally, reduced eNOS is associated with changes in these miRNAs. Therefore, miRNAs could play a critical role in arterial EC senescence [47]. In addition to suppressing eNOS, the increased miR-221/222 cluster inhibits their proangiogenic activation, proliferation, and migration in senescent HAECs [48].

Studies have found that many miRNAs are involved in the RS of ECs. For example, miR-92a is upregulated in the aged vascular endothelium, and oxidative stress or inflammation can be suppressed by inhibiting miR-92a expression and regulating the Nrf2-Keap1-antioxidant response element (ARE) signaling pathway, thus inhibiting endothelial apoptosis and facilitating cell proliferation; thus, the upregulation of miR-92a promotes cell apoptosis in the aged vascular endothelium [49]. However, miR-92a was downregulated in senescent HAECs, which inhibited cell proliferation [47]. Additionally, miR-21 was inconsistently expressed in different senescent ECs. miR-21 was also found to be upregulated in response to replicative and stress-induced senescence, and its overexpression induced p21^CIP1^ and pCDK2 expression by targeting CDC25A and nuclear factor 1B (NF-IB), thus promoting EC senescence [50]. However, the downregulation of miR-21 promoted apoptosis in senescent HAECs [47].

miR-126 is another controversial molecule in endothelial senescence. It was found that the downregulation of miR-126 in senescent human umbilical vein endothelial cells (HUVECs) reduced tube formation and wound healing closure by inhibiting hypoxia-inducible factor-1*α* (HIF-1*α*) expression [51]. However, in another study, miR-126-3p levels showed a significant age-related increase in senescent HUVECs. miR-126-3p overexpression enhances cell survival by targeting SPRED-1 and activating the phosphatidyl inositol 3-kinase (PI3K)/AKT/eNOS signaling pathway, which are involved in promoting cell differentiation and survival [52]. More research is required to determine what role these miRNAs play in endothelial senescence.

In ECs undergoing RS, miR-146a, miR-34a, and miR-181a are overexpressed, whereas their target, Bcl-2, an antioxidant and antiapoptotic factor that regulates mitochondrial fission/fusion and autophagy, is downregulated, leading to the accumulation of dysfunctional mitochondria, oxidative stress, chronic low-grade inflammation, and increased apoptosis [53]. Interestingly, another study found that miR-146a was downregulated in senescent HUVECs. Moreover, miR-146 affects EC senescence by targeting NOX4, a main source of reactive oxygen species (ROS). Therefore, the downregulation of miR-146a promotes EC senescence by increasing ROS [54, 55]. In addition, toll-like receptor (TLR) signaling-associated IL-1 receptor- associated kinase (IRAK1), a key mediator of the TLR/IL-1R signaling pathways that leads to the induction of inflammatory target gene expression, is another target of miR-146a [56]. Thus, the downregulation of miR-146a can also promote the expression of inflammatory genes in ECs by increasing the expression of IRAK1 in senescent HUVECs. It is difficult to explain the inconsistencies associated with the roles of miR-146a in HUVEC senescence, but it was confirmed that miR-146a is involved in EC senescence.

Further, miR-34a expression was increased in senescent HUVECs, and its overexpression was shown to induce EC senescence and suppresses cell proliferation by inhibiting sirtuin 1 (SIRT1) expression [57]. Moreover, the inhibition of SIRT1, an NAD^+^-dependent protein deacetylase, induces a premature senescence-like phenotype by increasing the acetylation of p53 and plasminogen activator inhibitor-1 (PAI-1) expression and decreasing both the protein expression and activity of eNOS [58]. P53 can also increase the expression of miR-34a in ECs [59]. This increase can induce senescence and vascular aging via the downregulation of its direct target gene *Sirt1*, which increases the acetylation of p53 and further increases the expression of miR-34a. Thus, the miR-34a/SIRT1/p53/miR-34a cycle promotes a positive feedback loop of EC senescence. In addition, miR-34a also induces endothelial progenitor cell (EPC) senescence by inhibiting SIRT1 to increase the expression of deacetylated forkhead box O1 (FoxO1), leading to impaired angiogenesis [60]. Moreover, miR-217 induces a premature senescence-like phenotype and leads to impaired angiogenesis via the inhibition of SIRT1 and modulation of FoxO1, as well as eNOS acetylation, in ECs [61].

miR-216a expression is significantly increased in senescent ECs and induces a premature senescence-like phenotype in HUVECs that is associated with impaired proliferation and migration and increased adhesion to monocytes; this is mediated by the inhibition of Smad3 expression and consequently the modulation of NF-*κ*B inhibitor alpha (I*κ*B*α*) degradation and adhesion molecule activation [62]. miR-216a promotes endothelial senescence and inflammation as an endogenous inhibitor of the Smad3/I*κ*B*α* pathway [62]. In addition, miR-22 was found to be upregulated in senescent EPCs. Accordingly, the overexpression of miR-22 in young EPCs induced cell senescence, decreased proliferation and migration, and impaired angiogenesis by sponging AKT3 (also known as protein kinase B3 (PKB3)) [63]. AKT3, one of the three AKT subtypes, is a serine/threonine kinase that promotes cell survival signals through the PI3K pathway, leading to the inactivation of apoptotic proteins [64]. miR-125a-5p expression was found to be upregulated in senescent arterial ECs, resulting in impaired angiogenesis through the targeting of RTEF-1 and the downregulation of eNOS and vascular endothelial growth factor (VEGF) [65].

Interestingly, the opposite is true for stress-induced EC senescence. Specifically, the expression of miR-125a-5p is decreased in oxidized low-density lipoprotein- (ox-LDL-) treated human brain microvascular endothelial cells (HBMECs). A further study found that miR-125a-5p overexpression could inhibit HBMEC senescence while promoting NO generation and reducing ROS production via PI3K/AKT/eNOS signaling [66]. These results suggest that miR-125a-5p plays a regulatory role through different signaling pathways during adaptation to different aging stresses. miR-299-3p is upregulated in senescent HUVECs, and one of its target genes could be insulin-like growth factor-1 (IGF1). Further, the knockdown of hsa-miR-299-3p was found to rescue cells from senescence induced by H_2_O_2_ treatment [67].

miR-10A∗ and miR-21 are upregulated in aged mice; Hmga2 is a shared molecular target of these miRNAs and a critical regulator of EPC senescence. The overexpression of miR-10A∗ and miR-21 in young EPCs causes EPC senescence, decreases self-renewal potential, increases p16^Ink4a^/p19^Arf^ expression by inhibiting Hmga2 expression, and eventually results in impaired EPC angiogenesis [68]. Further, miR-126, miR-21, and miR-100 levels were increased in senescent HUVECs, which decreased the glycolysis rate and reduced stress tolerance by targeting nuclear factor E2-related factor 2 (NRF2), a key antiaging transcription factor regulating oxidative stress responses and angiogenic capacity [69–71]. Furthermore, the upregulation of miR-144 in aged CMVECs also decreased the expression of NRF2, leading to increased age-related oxidative stress and impaired angiogenesis [72].

The miR-17-92 cluster encodes seven mature miRNAs: miR-17-5p, miR-17-3p, miR-18a, miR-19a, miR-20a, miR-19b, and miR-92a. One study found that miR-17, miR19b, miR-20a, and miR-106a were downregulated in senescent HUVECs and that a decrease in these miRNAs was correlated with increased transcript levels of CDK inhibitor p21/CDKN1A, establishing these miRNAs as novel markers of cellular aging in humans [73]. Another study found that miR-18a expression was decreased in aging ECs and miR-18a protects ECs from hypoxia/reoxygenation-induced injury by downregulating the Nox2/ROS pathway [74]. In addition, some components of the miR-17-92 cluster (miR-18a, miR-17-5p, and miR-20a) may participate in the control of angiogenic phenotypes such as the proliferation, survival, and organization of ECs [75]. Further, miR-214, which is highly expressed in ECs, is enriched in EC-derived exosomes; senescent cells with reduced miR-214 levels can be rescued by absorbing exosomal miR-214 produced by neighboring cells. Finally, miR-214 represses senescence by suppressing ATM, which prevents cell cycle progression [76].

Many miRNAs also play a regulatory role in SIPS in ECs. For example, radiation-induced miR-494 expression exacerbates DNA damage and drives endothelial senescence, which affects telomerase activity, activates p21, inhibits the pRB pathway, and diminishes angiogenic sprouting by targeting the MRE11a-RAD50-NBN complex [77].

miR-21-5p and miR-203a-3p are upregulated during ox-LDL-induced HUVEC senescence. Further studies have demonstrated that miR-21-5p/203a-3p promotes ox-LDL-induced EC senescence through the downregulation of dynamin-related protein 1 (Drp1), resulting in imbalances in mitochondrial dynamics, mitochondrial dysfunction, and activation of the AMP-activated protein kinase (AMPK)-p53/p16 pathway [78, 79]. miR-20b, a paralog of the miR-17-92 cluster, is upregulated during tumor necrosis factor-*α*-- (TNF-*α*-) induced premature senescence. Further, the knockdown of hsa-miR-20b was found to attenuate premature senescence in TNF-*α*-treated human pulmonary microvascular ECs by increasing target *RBL1* mRNA expression, but decreasing the protein expression of p16^INK4a^[80].

The miRNA-200 gene family consists of five members: miR-200a, miR-200b, miR-200c, miR-141, and miR-429. miR-200c was determined to be upregulated when HUVECs were exposed to H_2_O_2_; it was further shown to inhibit cell proliferation due to senescence. ZEB1 is a target of miR-200c, and its downregulation plays a key role in ROS-induced apoptosis and senescence. Further, pRB and p53 play an active role in the upregulation of miRNA-200c and EC senescence induced by H_2_O_2_ [81]. The expression of miR-200b was shown to be related to an aging-associated increase in EPCs; furthermore, research has found that miR-200b regulates apoptosis and senescence by suppressing c-Jun expression, thus negatively affecting the vascular repair capacity of the cells in atherogenesis [82]. miR-200a, another member of the miRNA-200 family, is increased in the corpus cavernosum of aged rats with erectile dysfunction (ED) compared to that in young rats with ED. In addition, the upregulation of miR-200a is involved in the mechanism underlying age-related ED via SIRT1 inhibition and the attenuation of endothelial function through its ability to influence the eNOS/NO/cGMP-dependent protein kinase (PKG) pathway [83].

Let-7g expression is also decreased in Ang II-induced EC senescence, whereas Let-7g overexpression reverses EC senescence [84]. Another study found that this molecule can improve several endothelial functions, including decreases in senescence, inflammation, and monocyte adhesion, as well as increases in angiogenesis, via transforming growth factor-*β* (TGF-*β*), and SIRT-1 signaling by regulating three target genes (*THBS1*, *TGFBR1*, and *SMAD2*) involved in TGF-*β* signaling and indirectly increasing *SIRT-1* expression to prevent EC senescence [85]. These studies suggest that miRNAs are involved in the regulation of EC senescence and promote senescent phenotypes.

### 4.2. miRNAs and VSMC Senescence

VSMCs also play an important role in vascular physiology. VSMC senescence leads to cell dysfunction and promotes the occurrence of diseases related to vascular aging. Accordingly, accumulating evidence indicates that miRNAs are involved in the regulation of VSMC senescence. For example, miR-34a is highly expressed in the aortas of old mice. Furthermore, miR-34a overexpression in proliferative human aortic smooth muscle cells causes cell cycle arrest in the G0–G1 phase along with enhanced p21 protein levels by targeting SIRT1 and stimulating the induction of proinflammatory factors including interleukin-1*β* (IL-1*β*), IL-8, IL-6, and bone morphogenic protein 2 (BMP2), as well as the chemokine monocyte chemoattractant protein 1 (MCP1) and the intercellular adhesion molecule 1 (ICAM1) [86]. In addition, miR-34a promotes vascular calcification via VSMC mineralization by inhibiting cell proliferation and inducing senescence [87]. miR-34a plays an important role in the senescence of both ECs and VSMCs and shows potential as a therapeutic target for antivascular aging strategies. Moreover, miR-30a is upregulated in senescent VSMCs, and rapamycin can alleviate aging VSMC cycle arrest and inhibit the senescence of these cells by downregulating miR-30a, which results in the upregulation of Beclin1 and the activation of autophagy [88].

Further, miR-92a expression is known to be reduced in the arteries of older adults. This reduction increases the arterial expression of type 1 collagen and tumor necrosis factor receptor 1 (TNFR1), which results in arterial dysfunction characterized by impaired carotid artery endothelium-dependent dilation and reduced NO bioavailability, as well as increased aortic stiffness and PWV [89]. However, miR-92a is upregulated during vascular EC senescence and is known to promote EC apoptosis [48]. Therefore, the inconsistent expression of miR-92a in senescent ECs and VSMCs might be related to different cell types. Nonetheless, this molecule is involved in the regulation of vascular aging.

miR-143 was found to be significantly upregulated in conjunction with the inhibition of AKT signaling in senescent VSMCs induced by H_2_O_2_. In addition, myocyte enhancer 2A (MEF2A) promotes the expression of miR-143 via Krüppel-like factor 2 (KLF2). Therefore, the MEF2A/miR-143/AKT pathway promotes aging in VSMCs [90].

miR-181b, another member of the miR-181 family, is decreased in the aortas of older mice, which increases the expression of targets such as TGF-*β*i (TGF-*β* induced) in the aortic VSMCs, thus promoting an increase in PWV, blood pressure, and vascular stiffness mediated by TGF-*β* signaling [91]. However, miR-181a was found to be overexpressed in senescent ECs. Further, the discordant expression of miR-181a and miR-181b is another interesting concept relating to vascular aging.

Aging is known to increase the expression of miR-203, which leads to a decrease in Src, Cav-1, and paxillin, which impairs agonist-induced focal adhesion signaling in aortic smooth muscle cells and increases VSMC stiffness in aortic tissues [92]. miR-135a was also found to be significantly decreased in aging VSMCs, and its inhibition markedly promoted cell calcification and the expression of calcification genes including *ALP* and *Osteocalcin* by targeting KLF4 and the KLF4/STAT3 pathway [93].

In addition to VSMCs, the extracellular matrix (ECM) is an important component in the aortic vascular media, and perturbations in ECM deposition are often associated with aging and aneurysms. miR-29 is significantly upregulated in aging arteries, mediating the downregulation of ECM proteins and sensitizing the aorta to the formation of aneurysms [94].

(↑) and (↓) indicate increased and decreased expressions, respectively, during senescence. (+) and (-) indicate the promotion or inhibition of senescence, respectively, by miRNA. N/A: not available.

### 4.3. lncRNAs and EC Senescence

In addition to miRNAs, lncRNAs have also been found to play an important regulatory role in vascular aging (Table 2). Using RNA-seq, Abdelmohsen et al. found that several lncRNAs were differentially expressed in senescent human fibroblasts compared to their expression in proliferating cells. Three random senescence-associated lncRNAs (SAL-RNAs) were found to affect the cell fate, and two of them (SAL-RNA2 and SAL-RNA3) were found to be related to cell survival; in contrast, SAL-RNA1 delays cell senescence, which suggests that lncRNAs play an important role in cell cycle control and senescence [95].

Some lncRNAs also participate in EC and VSMC aging, ultimately leading to a vascular aging phenotype. In a study of the aging mechanisms of EPCs, it was found that the overexpression of nicotinamide phosphoribosyltransferase (NAMPT) could promote cell proliferation and inhibit EPC senescence. Further, miR-223 was found to bind and inhibit the expression of NAMPT, whereas lncRNA GAS5 was suggested to alleviate NAMPT inhibition by sponging miR-223. Moreover, GAS5 knockdown remarkably downregulated cell activity and DNA synthesis in EPCs and promoted EPC senescence. Accordingly, miR-223 inhibition could partially attenuate the effects of GAS5 knockdown on NAMPT and EPC senescence. A further study found that the lncRNA GAS5/miR-223/NAMPT axis serves as a critical regulator of EPC proliferation and senescence via PI3K/AKT signaling [96]. NAMPT expression is decreased significantly in aging EPCs, whereas NAMPT overexpression can rescue these cells from senescence [96]. A subsequent study found that NAMPT upregulates the expression of SIRT1 AS lncRNA, which relieves miR-22-induced SIRT1 downregulation by competitively sponging miR-22 in EPCs. Therefore, NAMPT inhibits EPC senescence through a SIRT1 AS lncRNA/miR-22/SIRT1 pathway and promotes EPC proliferation and migration [97]. In addition, Visfatin was shown to alleviate ox-LDL-induced EPC senescence by inducing SIRT1 expression in EPCs. Further studies showed that SIRT1 upregulation can inhibit EPC senescence through the PI3K/Akt/ERK pathway [98].

Interestingly, the GAS5/miR-223/NAMPT/SIRT1 AS lncRNA/miR-22/SIRT1 axis and PI3K/Akt/ERK signaling pathway both play a regulatory role in EPC senescence. The expression of H19 is decreased in the endothelium of aging mice, and the loss of this marker results in the upregulation of p16 and p21, which inhibit proliferation and budding and increase EC senescence. Moreover, the deletion of H19 was shown to increase STAT3 phosphorylation and promote the expression of IL-6 and IL-6R*α*. Accordingly, the inhibition of STAT3 activation alleviates the effect of H19 silencing on the expression of p21 and vascular cell adhesion molecule-1. Thus, H19 depletion results in premature senescence and the dysfunction of ECs and induces inflammation via STAT3 signaling in these cells [99]. In addition, it was found that STAT3 induces p21 expression via the transcriptional activation of FoxP3 and by directly binding the *p21* gene promoter region [100, 101].

The lncRNA maternally expressed gene 3 (MEG3) in blood vessel specimens of aged individuals and mice was shown to be decreased significantly. The decline in the competitive adsorption of MEG3 leads to increased miR-128 expression and decreased Girdin expression, leading to HUVEC senescence and a reduction in platelet phagocytosis in HUVECs [102]. Interestingly, another study found that Meg3 was significantly increased in senescent HUVECs, and that the silencing of Meg3 prevented the aging-mediated inhibition of sprouting activity in HUVECs, which may be involved in Meg3-mediated changes in the epigenetic regulation of gene expression [103]. Further, the mitochondrial lncRNA ASncmtRNA-2 was found to be upregulated in the aorta of aged mice and replicative senescent ECs, but not in SIPS induced by ultraviolet light or H_2_O_2_ or in VSMCs, suggesting that its expression might be related to telomere length and cell type specificity. Further studies revealed that ASncmtRNA-2 might be a noncanonical precursor of hsa-miR-4485 and hsa-miR-1973. ASncmtRNA-2 can also promote cell cycle arrest at the G2/M phase and cell senescence by producing hsa-miR-4485 and hsa-miR-1973 [104]. hsa-miR-4485 interfered with 16S rRNA processing and decreased mitochondrial protein synthesis, leading to bioenergetic dysfunction and increased caspases-3/7 activity, which is a known hallmark of apoptosis induced by mitochondrial dysfunction [105]. These studies suggest that lncRNAs are involved in regulating EC senescence and senescence-related dysfunction through a variety of pathways.

The permanent inhibition of cell proliferation is considered a traditional marker of cell senescence; thus, some lncRNAs that inhibit cell proliferation might also be involved in cell senescence (Table 3). For example, lncRNA-ATB is increased in HUVECs treated with TGF-*β*1, which reduces HUVEC viability and proliferation. Furthermore, lncRNA-ATB overexpression upregulates caspase-3 in HUVECs and promotes atherosclerosis [106]. Additionally, HIF1A-AS1 was found to be upregulated in HUVECs induced by palmitic acid (PA). Moreover, HIF1A-AS1 silencing can reduce PA-induced apoptosis and promote the proliferation of HUVECs. Clopidogrel can also reduce PA-induced apoptosis and promote the proliferation of HUVECs by inhibiting HIF1A-AS1, thereby playing a cardioprotective role [107]. IGF2AS, a natural antisense RNA of IGF2, was found to be upregulated in diabetic myocardial microvascular endothelial cells (mMVEs), whereas the inhibition of IGF2AS upregulated IGF2 and VEGF, promoting the proliferation and invasion of diabetic mMVEs. Hence, IGF2AS might also be involved in angiopathy in type 2 diabetes mellitus [108].

LINC00305 expression is significantly upregulated in response to hypoxia in HUVECs. LINC00305 overexpression suppresses proliferation and enhances apoptosis in HUVECs by sponging miR-136, whereas LINC00305 downregulation has the opposite effects, suggesting that it can promote apoptosis and inhibit proliferation in these cells in response to hypoxia [109].

lncRNA OIP5-AS1 was also found to be significantly overexpressed in HUVECs administered with ox-LDL. Silencing this molecule inhibited apoptosis and promoted proliferation by inducing G0/G1 cycle arrest. A further study found that OIP5-AS1 reduces GSK-3*β* expression by recruiting EZH2, a critical element of the polycomb repressive complex 2 (PRC2) that directly binds the *GSK-3β* promoter region [110]. Moreover, lncRNA PINC was found to overexpressed in HUVECs treated with TNF-*α*. PINC knockout promotes the proliferation and inhibits the apoptosis of HUVECs. Therefore, TNF-*α* might partially induce the apoptosis of vascular ECs via PINC overexpression [111].

Finally, SNHG7 was found to be decreased in human retinal ECs exposed to high-glucose (HG) stimuli. SNHG7 overexpression suppresses the inhibition of SIRT by directly inhibiting miR-543, thereby reducing HG-induced cell proliferation, migration, angiogenesis, and VEGF expression [112]. Thus, SNHG7 is a potential molecular target to attenuate HG-induced angiogenesis through the miR-543/SIRT1/VEGF pathway.

In summary, these lncRNAs inhibit the proliferation of ECs through many mechanisms. The above lncRNAs are only a portion of the many lncRNAs involved in endothelial cell proliferation inhibition, but whether proliferation inhibition is caused by cell cycle arrest and cell senescence requires further study.

(↑) and (↓) indicate increased and decreased expressions, respectively, during senescence. (+) and (-) indicate the promotion and inhibition of senescence, respectively, by lncRNAs. N/A: not available.

### 4.4. lncRNAs and VSMC Senescence

Many studies have found that lncRNAs are also involved in the regulation of VSMC senescence. For example, antisense noncoding RNA in the *INK4* locus (ANRIL), a lncRNA encoded in the chromosome 9p21 region, regulates its neighbor, tumor suppressor CDKN2A/B, via epigenetic mechanisms and thereby regulates cell proliferation and senescence [113]. ANRIL and Sirt1 were found to be downregulated, whereas miR-181a was upregulated, in aging VSMCs. The overexpression of ANRIL can also promote cell viability and inhibit the senescence of VSMCs by directly regulating the expression of miR-181a and alleviating the inhibitory effect of Sirt1, further inhibiting the aging-induced activation of p53/p21 signaling [114]. Therefore, ANRIL downregulation promotes VSMC senescence through the miR-181a/Sirt1/p53/p21 pathway.

Growth inhibition specificity 5 (GAS5) is a well-known tumor suppressor lncRNA [115]. GAS5 expression is downregulated in proliferative VSMCs, and its overexpression induces cell cycle arrest at the G0/G1 phase through the p53 pathway, which is associated with cell senescence, inhibiting VSMC proliferation and promoting apoptosis. Regarding its underlying mechanism of action, GAS5 binds p53 and the p53 activator P300 to enhance the stability and activity of p53, resulting in the increased expression of cell cycle inhibitors and apoptosis activation genes, leading to cell cycle arrest and increased apoptosis in VSMCs. Moreover, the overexpression of GAS5 via adenoviral delivery was found to suppress neointima formation in a rat carotid balloon injury model, which was related to an increase in p53 expression and apoptosis in neointimal VSMCs [116]. GAS5 reduces the restenosis of balloon-dilated vessels and is also significantly downregulated in response to hypertensive stress. GAS5 knockdown increases proliferation, migration, and phenotypic switching in VSMCs. Mechanistically, it interacts with *β*-catenin and its dysregulation affects *β*-catenin nuclear translocation, ultimately altering *β*-catenin signaling [117].

The downregulation of GAS5 promotes VSMC proliferation and vascular remodeling, leading to hypertension. Vascular calcification is a prominent feature of arterial aging [118]. In a study on the calcification/senescence of HA-VSMCs induced by HG, it was found that lncRNA-ES3 is markedly increased in HA-VSMCs treated with HG, which alleviates the inhibition of BCL-2-modifying factor (BMF), promoting the calcification/senescence of HA-VSMCs by sponging miR-34c-5p. Further, lncRAN-ES3 acts as a competing endogenous RNA (ceRNA) of miR-34c-5p to regulate the expression of BMF in HA-VSMCs. BMF is a member of the proapoptotic Bcl-2 family, which is mainly associated with cell proliferation and apoptosis [119].

Some lncRNAs that inhibit the proliferation of VSMCs might also be involved in cell senescence. For example, HIF1A-AS1 expression is increased in intracranial aneurysms, and its overexpression increases the expression of TGF-*β*1 and inhibits the proliferation of VSMCs. Therefore, HIF1A-AS1 regulates the proliferation of VSMCs by upregulating TGF-*β*1 and participating in the development of intracranial aneurysms [120]. HIF1A-AS1 expression is also increased in thoracic aortic aneurysm (TAA) and is positively regulated by BRG1. HIF1A-AS1 silencing suppresses apoptosis and promotes the proliferation of VSMCs. Thus, BRG1 promotes apoptosis and inhibits the proliferation of VSMCs through the mediator HIF1A-AS1 [121].

lincRNA-p21 in aortic media tissues and blood is significantly upregulated in TAA patients. Further, lincRNA-p21 overexpression inhibits proliferation and promotes apoptosis in VSMCs through the activation of TGF-*β*1 signaling [122], and this marker is dramatically increased in atherosclerotic plaques. lincRNA-p21 represses cell proliferation and induces apoptosis in VSMCs by enhancing p53 transcriptional activity. A further study found that lincRNA-p21 binds directly to MDM2, resulting in the release of p53 from MDM2 and its subsequent binding to p300, which enhances the activity of p53, thereby inhibiting VSMCs proliferation [123]. MEG8 is downregulated in ox-LDL-treated VSMCs, and its overexpression suppresses cell proliferation and migration and induces apoptosis. Further research has found that MEG8 promotes the expression of peroxisome proliferator-activated receptor *α* (PPAR*α*) by sponging miR-181a-5p. PPAR*α* is a member of the PPAR family and promotes the degradation of cyclin-dependent kinase inhibitors (CDKIs) after its activation. Hence, MEG8 regulates the proliferation and migration of VSMCs via the MEG8/miR-181a/PPAR*α* axis [124].

lncRNA CASC11 is an oncogene in several types of cancer [125]. Its overexpression represses the proliferation and promotes the apoptosis of VSMCs. Accordingly, the downregulation of CASC11 in the plasma of atherosclerosis patients was found to promote VSMC proliferation and the expression of IL-9, which contributes to atherosclerosis [126]. Further, lncRNA MRAK048635 P1 exhibits low expression during hypertension and decreases its expression, promotes proliferation and migration, and inhibits apoptosis in VSMCs; this is a potentially important factor for vascular remodeling, as it affects VSMC cell function and phenotypic switching in essential hypertension [127]. These lncRNAs are representative of many lncRNAs involved in VSMC proliferation inhibition; however, whether these lncRNAs inhibit VSMC proliferation by promoting cell cycle arrest and senescence requires further study.

## 5. Conclusions

Vascular aging inevitably occurs during the process of aging. The senescence of ECs and VSMCs is the major factor in vascular aging and is regulated by miRNAs and lncRNAs. Therefore, miRNAs and lncRNAs comprise potential therapeutic targets for many associated diseases and conditions. For example, the expression of miR-34 family members (miR-34a, miR-34b, and miR-34c) is elevated in heart disease, and the inhibition of these factors with anti-miR-34a/anti-miR-34 has emerged as a promising therapeutic strategy.

miR-29b-3p is highly expressed in the exosomes of bone marrow mesenchymal stem cells (BM-MSCs) of aged mice, and the uptake of these exosomes by adipocytes, muscle cells, and hepatocytes leads to insulin resistance. However, an aptamer-mediated nanocomplex delivery system that downregulates miR-29b-3p in BM-MSC-derived exosomes was found to significantly ameliorate insulin resistance in aged mice [128]. Moreover, levels of miR-214 are significantly decreased in senescent ECs, whereas miR-214 produced by adjacent cells can play an antiaging role through exosomal incorporation into senescent cells [76]. As a membrane-bound vesicle secreted by cells, exosomes can transport ncRNAs to the target cells without being degraded by RNAse enzymes. After uptake, ncRNAs are released and play a regulatory role [129]. Therefore, it is possible that a combination of exosomes and ncRNAs could be used for the treatment of vascular aging. However, the expression of some miRNAs or lncRNAs is not consistent in vascular aging, and these interventions may have different results. Therefore, further studies are required for its future applications.

## Figures and Tables

**Table 1:** MicroRNAs in vascular aging.

MicroRNA	Cell type	Pathway	Effect on senescence	Functional consequences	Reference
miR-221/222 (↑)	HAEC	N/A	N/A	Reduced eNOS, inhibited proliferation, migration, and angiogenesis	[47, 48]
miR-92a (↓)	HAEC	N/A	N/A	Inhibited proliferation	[47]
miR-92a (↑)	HUVEC	Nrf2-KEAP1-ARE	N/A	Promoted apoptosis	[49]
miR-21 (↓)	HAEC	N/A	N/A	Promoted apoptosis	[47]
miR-21 (↑)	HUVECs	Targeting CDC25A and NFIB	**±**	Inhibited angiogenesis and proliferation	[50]
miR-126 (↓)	HUVEC	HIF-1*α*	N/A	Inhibited migration, proliferation, and angiogenesis	[51]
miR-126 (↑)	HUVEC	SPRED-1	N/A	Promoted differentiation and survival	[52]
miR-181a (↑)	HUVECs	Targeting Bcl-2	N/A	Promoted oxidative stress, chronic low-grade inflammation, and apoptosis	[53]
miR-146a (↑)	HUVECs	Targeting Bcl-2	N/A	Promoted oxidative stress, chronic low-grade inflammation, and apoptosis	[53]
miR-146a (↓)	HUVECs	Targeting NOX4 and IRAK1	—	Increased ROS and promoted inflammation	[54–56]
miR-34a (↑)	HUVECs, EPCs	Targeting Bcl-2 and SIRT1	**+**	Promoted apoptosis and inflammation	[53, 57, 60]
miR-217 (↑)	HUVECs, HAEC	Targeting SIRT1	**+**	Inhibited angiogenesis	[61]
miR-216a (↑)	HUVECs	Smad3/I*κ*B*α*	**+**	Inhibited proliferation and migration, increased adhesion to monocytes	[62]
miR-22 (↑)	EPCs	Targeting AKT3	**+**	Inhibited proliferation, migration, and angiogenesis	[63]
miR-125a-5p (↑)	AEC	Targeting RTEF-1	N/A	Inhibited angiogenesis	[65]
miR-125a-5p (↓)	HBMEC	PI3K/Akt/eNOS	—	Promoted apoptosis and inhibited proliferation, migration, and angiogenesis	[66]
miR-299-3p (↑)	HUVECs	Targeting IGF1	**+**	Inhibited proliferation, migration	[67]
miR-10A∗/miR-21 (↑)	EPCs	Targeting Hmga2	**+**	Inhibited angiogenesis	[68]
miR-126/miR-21/miR-100 (↑)	HUVEC	Targeting NRF2	**+**	Decreased the glycolysis rate and stress tolerance	[69]
miR-144 (↑)	CMVEC	Targeting NRF2	**+**	Increased oxidative stress and inhibited angiogenesis	[71]
miR-17-92 (↓)	HUVEC	p21/CDKN1A	**—**	Inhibited proliferation, survival, and angiogenesis	[72–75]
miR-214 (↓)	HMVEC	Targeting ATM	—	Inhibited angiogenesis	[76]
miR-494 (↑)	HUVECs	Targeting MRN	**+**	Inhibited angiogenesis	[77]
miR-21-5p/203a-3p (↑)	HUVECs	Drp1/AMPK-p53/p16	**+**	Mitochondrial dysfunction	[78, 79]
miR-20b (↑)	HMVEC	Targeting RBL1	**+**	Inhibited proliferation	[80]
miR-200c (↑)	HUVECs	Targeting ZEB1	**+**	Inhibited proliferation	[81]
miR-200b (↑)	EPC	Targeting c-Jun	**+**	Promoted apoptosis	[82]
miR-200a (↑)	Cavernous ECs	SIRT1*/eNOS/NO/PKG*	**+**	Attenuated endothelial function	[83]
Let-7g (↓)	HUVECs	SIRT1/TGF-*β*	—	Increased inflammation, monocyte adhesion and decreased angiogenesis	[84, 85]
miR-34a (↑)	HASMCs	Targeting SIRT1	**+**	Promoted inflammation and vascular calcification, inhibited proliferation	[86, 87]
miR-30a (↑)	VSMCs	Targeting Beclin1	**+**	Inhibited autophagy	[88]
miR-92a (↓)	VSMCs	TNFR1	N/A	Promoted aortic stiffness	[89]
miR-143 (↑)	VSMCs	Targeting AKT	**+**	Inhibited proliferation, migration	[90]
miR-181b (↓)	VSMCs	TGF-*β*	N/A	Promoted vascular stiffness	[91]
miR-203 (↑)	Aortic SMCs	Targeting Src, caveolin-1 and paxillin	N/A	Promoted vascular stiffness	[92]
miR-135a (↓)	VSMCs	KLF4/STAT3	N/A	Promoted cell calcification	[93]

**Table 2:** lncRNAs in vascular aging.

lncRNA	Cell type	Pathway	Effect on senescence	Functional consequences	Reference
GAS5 (**↓)**	EPCs	miR-223/NAMPT and PI3K/AKT	—	Inhibited proliferation	[96]
SIRT1 AS (**↓)**	EPCs	miR-22/SIRT1 and PI3K/AKT/ERK	—	Inhibited proliferation and migration	[97, 98]
H19 (**↓)**	HUVECs	STAT3	—	Inhibited proliferation and angiogenesis, promoted inflammation	[99]
MEG3 (**↓)**	HUVECs	miR-128/Girdin	—	Inhibited platelet phagocytosis	[102]
MEG3 (**↑)**	HUVEC	N/A	+	Inhibited angiogenesis	[103]
ASncmtRNA-2 (**↑)**	HUVECs	miR-4485 and miR-1973, 16S rRNA	+	Promoted apoptosis	[104, 105]
ANRIL (**↓)**	VSMCs	miR-181a/SIRT1	—	Promoted cell viability	[114]
GAS5 (NA)	VSMCs	p53, P300, and *β*-catenin	N/A	Promoted apoptosis and inhibited proliferation, neointima formation	[116, 117]
ES3 (**↑)**	VSMCs	miR-34c-5p/BMF	+	Promoted calcification	[119]

**Table 3:** lncRNAs associated with proliferation inhibition of endothelial cells and vascular smooth muscle cells.

lncRNA	Cell type	Pathway	Functional consequences	Reference
ATB	HUVECs	Caspase-3	Inhibited proliferation, promoted apoptosis	[106]
HIF1A-AS1	HUVECs	N/A	Inhibited proliferation and promoted apoptosis	[107]
IGF2AS	mMVEs	IGF2/VEGF	Inhibited proliferation and invasion	[108]
LINC00305	HUVEC	Sponging miR-136	Inhibited proliferation and promoted apoptosis	[109]
OIP5-AS1	HUVEC	GSK-3*β*	Inhibited proliferation and promoted apoptosis	[110]
PINC	HUVEC	N/A	Inhibited proliferation and promoted apoptosis	[111]
SNHG7	hREC	miR543/SIRT1	Inhibited proliferation, migration and angiogenesis	[112]
GAS5	HUVECs、VSMCs	*β*-Catenin	Inhibited proliferation, migration, and phenotypic switching	[117]
HIF1A-AS1	VSMCs	TGF-*β*1	Inhibited proliferation and promoted apoptosis	[120, 121]
lincRNA-p21	VSMCs	TGF-*β*1, P53	Inhibited proliferation promoted apoptosis	[122, 123]
MEG8	VSMCs	miR-181a-5p/PPAR*α*	Inhibited proliferation and migration and induced apoptosis	[124]
CASC11	VSMCs	IL-9	Inhibited proliferation and promoted apoptosis	[126]
MRAK048635 P1	VSMCs	N/A	Inhibited proliferation, promoted apoptosis and phenotypic switching	[127]
